# COLLABORATING WITH LONG-TERM CARE PROVIDERS IN ELDER MISTREATMENT RESEARCH

**DOI:** 10.1093/geroni/igad104.0421

**Published:** 2023-12-21

**Authors:** Karl Pillemer, Rhoda Meeador, Leslie Schultz, Leann Rorick, Karen Robson, Jeanne Teresi

**Affiliations:** Cornell University, Ithaca, New York, United States; Cornell University, Ithaca, New York, United States; Cornell University, Ithaca, New York, United States; Lifespan of Greater Rochester, Rochester, New York, United States; PA Department of Military and Veterans Affairs, Scranton, Pennsylvania, United States; Columbia University Stroud Center, New York City, New York, United States

## Abstract

To study elder mistreatment in institutional settings, collaboration is needed with nursing home and assisted living providers. Studies show that common problems emerge in researcher-practitioner collaboration, including differences in the key areas of orientation, training, nature of evidence, time horizons, and uses of research. Barriers specific to collaboration with. LTC providers also present challenges, including reputational risk from participating in EM studies and demands on administrative and staff time that limit availability for research. We report on a successful model for collaboration with LTC providers, based on the implementation and evaluation of the “Improving Resident Relationships in Long-Term Care” program. This evidence-based program was implemented to prevent and treat resident-to-resident aggression in nursing homes (16 facilities) and assisted living (14 facilities), involving over 2000 residents. Findings revealed a set of replicable elements to obtain LTC provider collaboration on sensitive issues like resident-to-resident aggression: (1) Preparatory efforts to gain commitment of administrative personnel; (2) agreements regarding confidentiality and anonymity; (3) identification of one or more “facility champions,” who demonstrate enthusiasm and willingness to advocate for the program; (4) providing payment to participating facilities to cover expenses such as overtime needed to release staff to attend trainings; (5) gaining the support of trusted provider organizations; (6) demonstrating that the program helps meet regulatory requirements; and (7) a procedure to allow for pragmatic modifications to the intervention that allow greater fit with the provider’s workflow. Implications for elder mistreatment intervention research are discussed.

